# Acute Oral Mammalian Toxicity and Effect of Solvents on Efficacy of* Maerua edulis* (Gilg. & Ben.) De Wolf against* Rhipicephalus (Boophilus) decoloratus* Koch, 1844 (Acarina: Ixodidae), Tick Larvae

**DOI:** 10.1155/2016/7078029

**Published:** 2016-08-31

**Authors:** Emmanuel T. Nyahangare, Brighton M. Mvumi, Tatenda Maramba

**Affiliations:** ^1^Department of Animal Science, University of Zimbabwe, P.O. Box MP167, Mount Pleasant, Harare, Zimbabwe; ^2^Department of Soil Science & Agricultural Engineering, University of Zimbabwe, P.O. Box MP167, Mount Pleasant, Harare, Zimbabwe

## Abstract

Efficacy and toxicity of aqueous and organic solvents extracts of* Maerua edulis* against ticks and mice, respectively, were determined. Ground leaves were extracted separately using cold water, cold water plus surfactant (1% v/v liquid soap), hot water plus surfactant, hexane, or methanol to make 25% w/v stock solutions from which serial dilutions of 5, 10, 20, and 25% were made. For each concentration, 20* Rhipicephalus decoloratus* tick larvae were put in filter papers impregnated with extracts and incubated for 48 h at 27°C and 85–90% RH for mortality observation after 24 h and 48 h. In the toxicity experiment, hot water plus surfactant treatments of 5, 10, 20, and 25% (w/v)* M. edulis* were administered in suspension* per os* to sexually mature Balb/C mice and observed for clinical signs and mortality for 72 h. Larvae mortality was highest (>98%) in methanol-extracted* M. edulis* treatments (20 and 25%), which was not different from the amitraz-based control (Tickbuster®). Mortality was also higher in the hot water than in cold water plus surfactant treatments (*P* < 0.05). No postadministration adverse health effects were observed in the mice. These results suggest that* M. edulis *is an effective tick remedy best extracted using methanol or hot water plus surfactant.

## 1. Introduction

The control of ticks remains prohibitively costly with global estimates for cost of conventional tick control in excess of USD7 billion per year [1]. It is also estimated that losses associated with ticks and tick-borne diseases (TTBD) range between USD13.9 and USD18.7 billion [2]. As a result, there is continued demand for new and improved methods of controlling ticks with the general consensus being that current conventional tick control methods are inadequate for several reasons [3, 4] or inaccessible, especially in the context of smallholder farmers. Intensive and/or indiscriminate use of arsenic, organophosphorus, and pyrethroid acaricides has led to serious problems of development of resistance in ticks [5–7]. In most parts of the world, chlorinated hydrocarbon acaricides are no longer used or marketed due to high toxicity and long residual effect on meat and other animal products [4]. There are also concerns on effective disposal means of these chemicals and the negative effects on the environment. Meanwhile, the threat of ticks and tick-borne diseases (TTBD) to livestock productivity and farmers' livelihoods remains high, especially for resource-poor and marginalised farmers [8, 9]. Livestock, and especially cattle, have a direct correlation with livelihoods issues in developing countries such that when the productivity of animals is poor, the quality of life of many people also suffers [3].

The use of locally available plants that have acaricidal properties has been hypothesized as a potentially effective method for holistic management of TTBD, especially in developing countries with limited financial resources to purchase the synthetic acaricides [10]. However, Stevenson and coworkers [11] noted that the scientific chemical structures of such plants and their associated activities are still poorly understood, which prevents their effective use and gaining such knowledge will enhance their usefulness and optimal use. Examples of plants that possess acaricidal properties against cattle ticks include* Tephrosia vogelii* Hook.f. [12, 13],* Azadirachta indica* A Juss. [14–16],* Origanum minutiflorum* O. Schwarz et P.H. Davis [17],* Euphorbia ovalifolia* Klotzsch & Garcke,* Ficus brachypoda* (Miq.) Miq. [18],* Petiveria alliacea* L. [19], and* Copaifera reticulata* Ducke [20]. In Zimbabwe, Madzimure et al. in [21] showed that simple aqueous leaf crude extracts of* Lippia javanica* were effective against cattle ticks at 10% w/v application level. In laboratory assays and on-station cattle experiments,* Strychnos spinosa* Lam and* Solanum incanum* L. also demonstrated that they have antitick properties [22]. The confirmation of acaricidal properties in these plants has opened up interest in other plants that possess similar properties and in finding ways to increase the efficacy. Previous on-station studies showed that some plant-based treatments were not as effective as commercial synthetic acaricides [21, 22].

**Figure 1:**
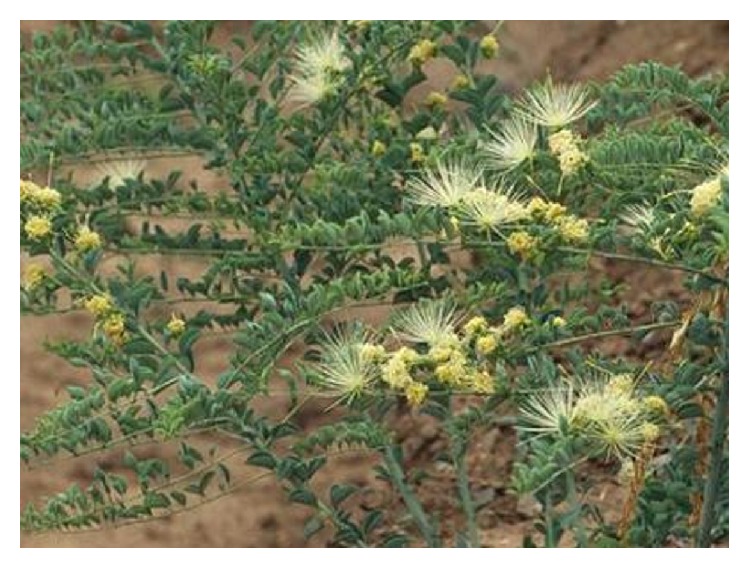
*Maerua edulis* plant in flower; picture courtesy of Aluka (http://www.bihrmann.com/caudiciforms/subs/mae-edu-sub.asp).

Blue bush-berry*, Maerua edulis* (Gilg. & Ben.) De Wolf (Figure 1), is a shrub that can be added to the database of plants with antitick properties. The plant is of the Capparaceae family found in some African countries including Democratic Republic of Congo, Kenya, Tanzania, Malawi, South Africa, Uganda, Zambia, and Zimbabwe in well drained soils [23].

Early efforts to validate the efficacy claims of* M. edulis* against cattle ticks were reported by Kaposhi et al. in [24] where results showed some antitick properties in the aqueous extracts of the plant. Apart from uses in tick control, the plant has other uses in grain protection during storage in some areas of Zimbabwe, particularly in and around Gokwe and Binga districts [25]. Despite the pesticidal properties, there have not been follow-up studies to further investigate and optimize the reported activity. In the current study,* M. edulis* was tested for efficacy against ticks using different extraction solvents as an optimization strategy. Nontarget safety was also evaluated in laboratory tests by administering extracts in single dose acute oral toxicity studies using Balb/C mice.

## 2. Materials and Methods

Laboratory experiments were conducted to determine the effect of different extraction solvents on the efficacy of* M. edulis* against* R. decoloratus* tick larvae (Experiment I) and to determine the relative safety of* M. edulis* on Balb/c mice (Experiment II).

### 2.1. Experiment I: The Laboratory Efficacy of* M. edulis* Crude Extracts against* R. decoloratus* Tick Larvae

#### 2.1.1. Study Site

The experiments were conducted at the Central Veterinary Laboratory (CVL) of Zimbabwe in Harare.

#### 2.1.2. Tick Collection and Rearing

Adult fully engorged ticks with no history of acaricidal resistance were collected from cattle in Mutorashanga (17.1488°S, 30.6761°E), in Makonde district and Mashonaland West province. The area is in Natural farming region II, characterized by annual rainfall of values between 700 and 1050 mm and a mean maximum temperature range of 16–19°C [26]. The ticks were prepared as described by Madzimure et al. in [22] by experts at the Central Veterinary Laboratory (Department of Veterinary Services in the Ministry of Agriculture, Mechanisation and Irrigation Development, Zimbabwe). Initially, the ticks were cleaned of all possible eggs laid during transportation from the field to the laboratory using distilled water. Subsequently, the ticks were put in plastic rearing tubes firmly closed with a ventilated stopper for egg laying in an incubator set 27-28°C and 85–95% relative humidity. All eggs laid were collected within 7 days from commencement of incubation and hatched. Tick larvae between 17 and 21 days were used for the larval package test.

#### 2.1.3. Plant Material Collection and Preparation

Leaves of the* M. edulis* plant were collected from a remote district called Binga (17.6241°S, 27.3411°E) found in the northern low-veld of Zimbabwe with an altitude of about 500 m. The district is in natural farming region V, characterized by very low rainfall and high temperatures (<450 mm per annum and 30°C, resp.). The collected fresh* M. edulis* leaves were shade-dried under ambient mean temperatures ranging from 25 to 32°C after which they were ground to powder and stored in a freezer at temperatures below 5°C until point of use.

#### 2.1.4. Treatments

For all the treatments, 5 g of the* M. edulis* powder was weighed and soaked separately in 20 mL of each of the following solvents: cold water, hot water (at 70°C), hexane, and methanol for 24 h to produce a stock solution of 25% w/v. The mixture was filtered through a filter paper and serial dilutions of 5, 10, 20, and 25% v/v made with each solvent. 1% v/v of liquid soap was added to the initial extraction mixture producing the stock solution, where the effect of adding a surfactant was to be determined. The aqueous extraction experiments used distilled water and distilled water plus surfactant as the negative controls and the organic solvents used for extraction as the negative controls (hexane or methanol). A commercial amitraz-based acaricide (Tickbuster) was prepared as per manufacturer's specifications (0.2% v/v) and used as the positive control. A summary on how the treatments were prepared is presented in Table 1.

#### 2.1.5. Experimental Procedure

Different concentrations of* M. edulis* extracts and the negative and positive controls were tested on approximately 20, 2-3-week-old tick larvae, using an adaptation of the Soberanes bioassay technique described by Miller et al. in [27]. For each treatment 10 mL of the solution was placed into a 10 cm diameter Petri dish containing a 9 cm diameter Whatman number 1 filter paper for 5 minutes. The tick larvae were then placed onto the wet filter paper which was then folded and the open end was sealed with steel paper clips. The packets were put in an incubator set at 27°C, 85–90% RH, and at photoperiod of 12 : 12 (L : D). Larval mortality was recorded after 24 and 48 h after incubation. Each treatment was replicated five times. In previous experiments with* M. edulis* described by Kaposhi and coworkers in [24], the efficacy was tested using the larval immersion technique (LIT) and the exposure to treated surface method was described, respectively, in [28, 29]. Aspects of the Soberanes technique are not very different from the LIT.

#### 2.1.6. Statistical Analysis

Mortality data for the tick bioassays was converted to ranks through the Proc. Frequency and Proc. Rank procedures of [30] and analysed using the Proc. GLM procedures of SAS.

### 2.2. Experiment II: Single Dose Acute Oral Toxicity of* M. edulis* on Balb/C Mice

#### 2.2.1. Study Site and Plant Material Preparation

The acute oral toxicity experiment was carried out in the Department of Animal Science, Bioassay Laboratory, at the University of Zimbabwe. Hot water plus surfactant-extracted* M. edulis* was used in the toxicity study and the treatments (5, 10, 20, and 25% v/v) were prepared as in the efficacy experiments (Experiment I; Treatment 3 under Table 1). Hot water with a surfactant was used as the control.

#### 2.2.2. Animals and Experimental Design

A total of 30* BALB/c* mice, reared in individual cages from time of weaning until sexual maturity at six weeks, were tested in a completely randomized design experiment. The mice were randomly allocated to the five treatments, in which each mouse represented an experimental unit and replicated sixfold. The animals were fed commercial mouse comproids procured from National Foods (Pvt. Ltd., Harare, Zimbabwe) and water* ad libitum* until one day before administration of the plant extracts. All feed and water were removed and reintroduced shortly after treatment administration.

The mice husbandry and the toxicity tests were adapted from the Organization for Economic Cooperation and Materials (OECD) guidelines for assessing acute oral toxicity. The Department of Animal Science is licensed by the Veterinary Services Unit in the Ministry of Agriculture, Mechanisation and Irrigation Development in Zimbabwe under the Scientific Animal Experiments Act (License Number L624) to carry out such experiments with mice. The Ethics Committee of the University of Zimbabwe approved the experimental protocols.

#### 2.2.3. Administration of Plant Extracts

The* M. edulis* plant extracts (5, 10, 20, and 25% v/v) and the control (distilled water) were administered to mice in suspension* per os* using a 10 mL plastic syringe with a 16 mm long 22-inch gavage needle connected to it as described in [31]. For normal physiological functions, the minimum water requirements of a mouse are on average 4 mL/day depending on weight [32] and this was used as the basis for the quantity administered to the mice.

#### 2.2.4. Monitoring and Measurements

The mice were observed daily for 3 days, checking for mortality, behavioural changes, and development of any clinical signs.

## 3. Results

### 3.1. Experiment I: Efficacy of Differently Extracted* Maerua edulis* against* R. decoloratus* Tick Larvae

#### 3.1.1. Aqueous Solvents

**Figure 2:**
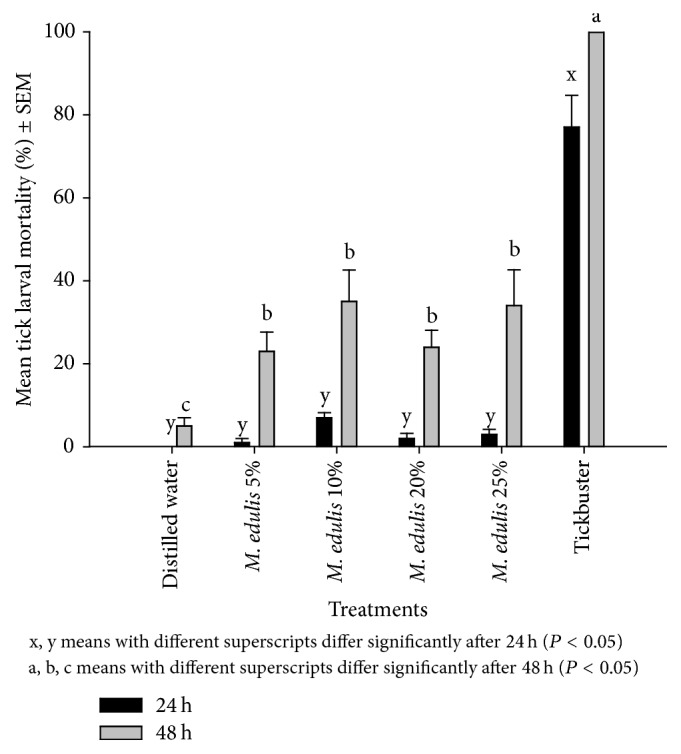
Effect of cold water extracts of* M. edulis* on* R. (B) decoloratus* tick larvae.

**Figure 3:**
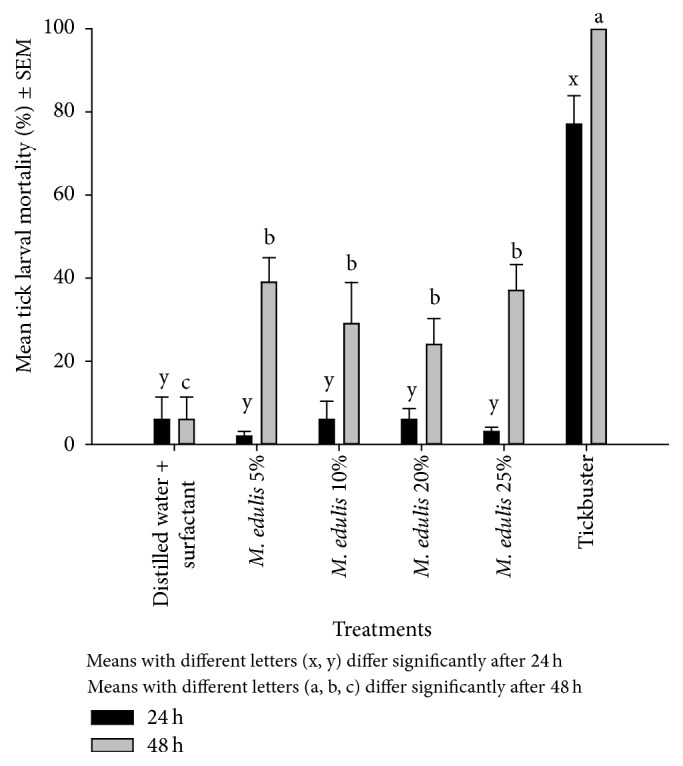
The effect of water plus 1% surfactant of* M. edulis* extracts on* R. (B) decoloratus* tick larvae.

Tick larvae mortality was significantly low (<40%) (*P* < 0.05) at both recording times in the* M. edulis* cold water only extractant and the negative control compared to the positive control (Tickbuster) which recorded 71% and 100% after 24 h and 48 h, respectively (Figure 2). In the cold water and surfactant treatments, larvae mortality was also generally low and below 80% for both sampling periods (Figure 3). The least square mean (LSM) mortalities were all significantly different to the Tickbuster positive control after 24 h and 48 h (*P* = 0.0001).

**Figure 4:**
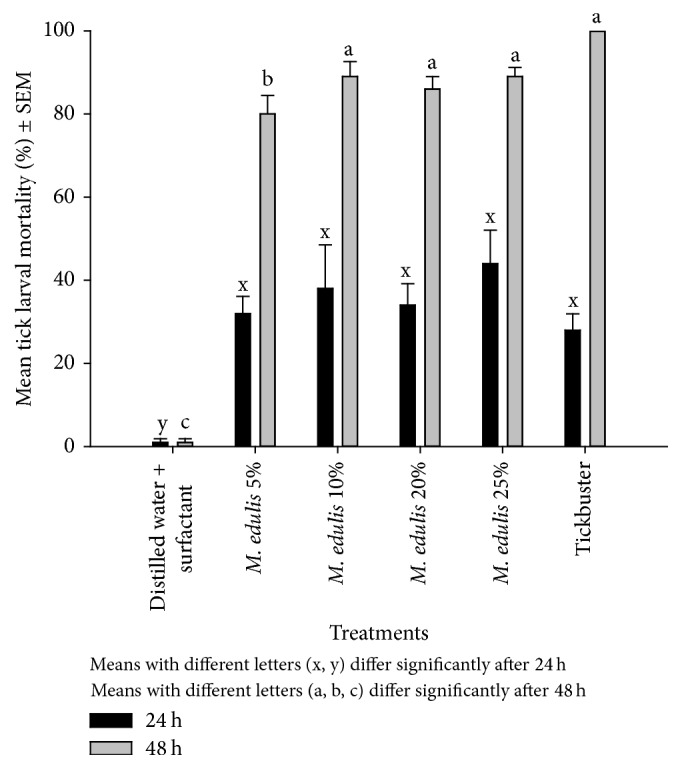
The effect of hot water and 1% surfactant-extracted* M. edulis* on* R. (B) decoloratus* tick larvae.

Larval mortalities in the* M. edulis* extract that used hot water and 1% surfactant were lower after 24 h but increased significantly after 48 h (Figure 4). There was no significant difference between the positive control and the plant extracts and between mortalities in the different plant concentrations (5, 10, 20, and 25%). Hot water extraction and the addition of a surfactant caused higher mortalities in the tick larvae compared to cold water treatments (Table 2).

#### 3.1.2. Organic Solvents

**Figure 5:**
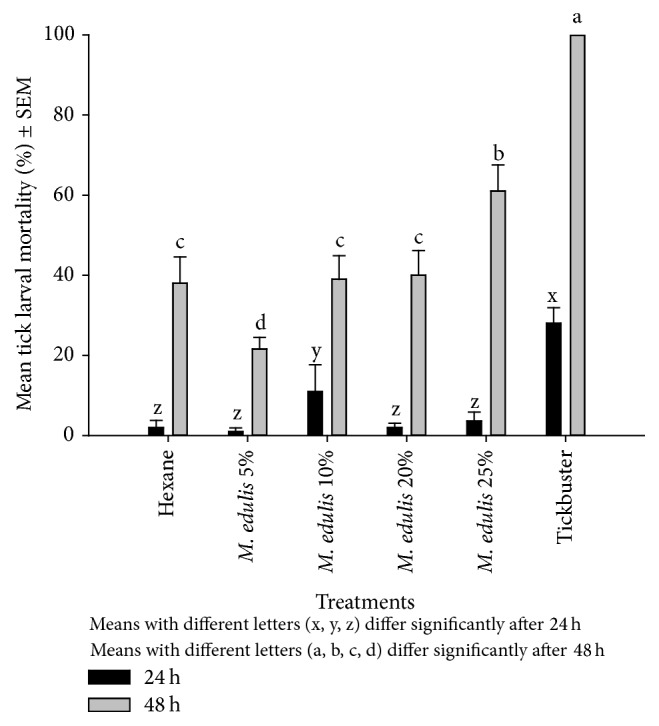
Effect of hexane extracted* M. edulis* on* R. (B) decoloratus* tick larvae.

There was no significant difference in mortality between the hexane control and hexane extracted* M. edulis* after 24 h. Even though the tick larval populations were reduced after 48 h, there was still no significant difference between mortality in the hexane control and hexane extracted plant extracts up to the 20% treatment (Figure 5). The mortality of the ticks was highest in the positive control (Tickbuster) than in all the other treatments (*P* < 0.0001).

**Figure 6:**
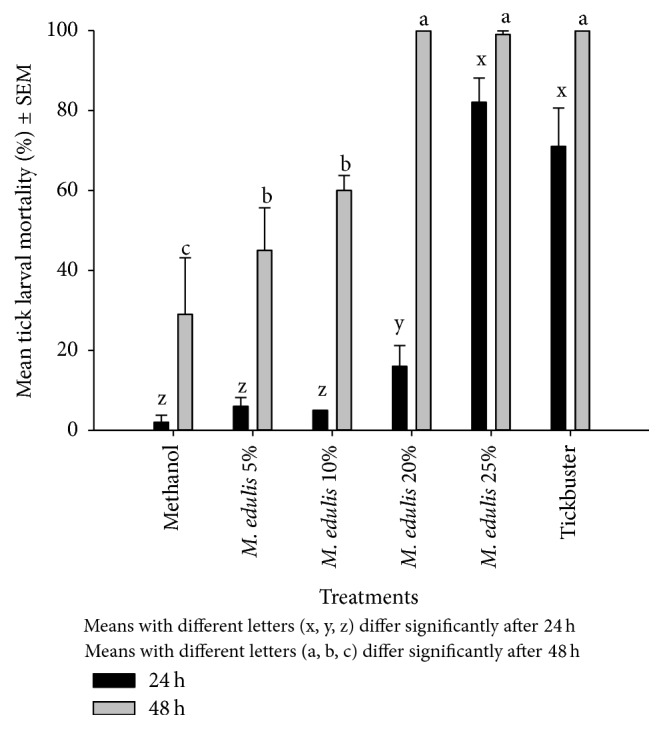
Effect of methanolic extracts of* M. edulis* against* R. (B) decoloratus* tick larvae.

In the methanol treatment, after 24 h, larvae mortality was low except in the 25% treatment, which recorded 80% mortality (Figure 6). There was dose-dependent increase in larval mortality after 48 h from 5–25% w/v* M. edulis* treatments. After 48 h, all* M. edulis* treatments, except the 5% treatment, showed significant mortality differences compared to the negative methanol control (*P* < 0.05), but it was only the 20 and 25% w/v treatments that had insignificant rank compared to the Tickbuster positive control.

### 3.2. Experiment II: Acute Oral Toxicity of* M. edulis* Extracts against Mice

The mice in all the treatments only appeared lethargic soon after oral administration but regained normal activity after 30–60 minutes. Neither obvious clinical signs nor mortality was recorded during the observation period.

## 4. Discussion

The study provides evidence that* M. edulis* does have antitick properties which are better expressed under hot water and organic solvent extraction. This is confirmation of work done earlier by Kaposhi reported in [24] wherein a modest efficacy of 51% was reported for aqueous extracts of this plant against* Rhipicephalus appendiculatus* tick larvae. Combined with other insecticidal properties against postharvest grain pests reported by Stathers et al. in [25],* M. edulis* can also control a wide array of agricultural pests. There is, however, more documented information on the medicinal properties compared to the pesticidal properties of the plant. This is synonymous with most plant materials used in ethnoveterinary practices where initial interest is for treatment of particular diseases, but because of the multifunctional nature of the plants, other uses are also discovered. The tubers of* M. edulis* have anthelminthic properties against livestock nematodes and have been used successfully against the faecal worm (*Heligmosomoides polygyrus*) eggs in sheep [33]. In Tanzania, Komwihangilo et al. in [34] reported the use of* M. edulis* and* Boscia grandiflora* leaves in the treatment of poultry diseases. In another study by [35], hexane extracts of* M. edulis* were observed to possess notable activity against* Mycobacterium bovis* and tuberculosis. Despite all this, there has not been many studies on the phytochemistry of* M. edulis* but hexane extracts of this plant have been reported to consist of linear chain unsaturated fatty acids which have been attributed to the observed biological activity [35].

The hot water with 1% surfactant extract of* M. edulis* gave better mortality results compared to cold water with or without a surfactant extract. Extraction of bioactive compounds is dependent on several factors including type of solvent and extraction method used [36]. While water is generally regarded as a safe universal solvent used for preparing traditional remedies, several authors have noted its limitation as a solvent because of high polarity [37–39]. Hot water however provides better results in the preparation of ethnomedicines and is widely used in countries where traditional medicines are commonly used like China and India [40, 41]. In South Africa and many other countries,* L. javanica* leaves are immersed in hot water for the efficient extraction of essential oils in the preparation of herbal tea for treatment of colds [42]. Increasing the temperature to a threshold level breaks the bonds tightly holding the water molecules together making it less polar. Surfactants are also known to increase the efficacy of traditional remedies [43, 44]. However, some surfactants can also cause toxicity to the ticks especially if they are in high concentrations as observed by Gonçalves et al. in [45]. The use of a detergent like liquid soap has been recommended and used by several authors as they work both as a surfactant and as a spreader at 1% v/v inclusion level [13, 39, 46].

After 48 h postincubation, hot water plant extracts performed as well as methanol extracts. This indicates that the yield of bioactive compounds extracted from the plant material by using hot water approaches that obtained by using methanol. Methanol-extracted* M. edulis* showed high acaricidal activity against tick larvae because the hydroxyl (-OH) on the methanol formula (CH_3_OH) contains a greater negative charge than the methane structure, making it a very effective solvent [47]. Elsewhere Afify et al. in [48] extracted* Syzygium cumini *L. using methanol, hexane, and ethyl acetate in experiments to determine acaricidal activity of the plant against the two-spotted spider mite,* Tetranychus urticae* Koch. The methanol extracts showed the highest acaricidal activity of 95.5% mortality, while hexane and ethyl acetate had 94% and 90% mortalities, respectively. The insignificant mortalities obtained by hexane only and hexane-extracted* M. edulis* are an indication that the mortality of the ticks could be coming from the hexane and not the active compounds from the* M. edulis*. The use of hexane and other solvents like acetone, chloroform, and butanol is not recommended in efficacy experiments because they have been shown to be very toxic especially to adult engorged ticks [49]. In this trial, hexane was however because tick larvae are not as susceptible to the toxicity of solvents compared to adult ticks as reported by Gonçalves et al. and Ravindran et al. in [45, 49]. Acetone, for example, one of the most toxic solvents in Adult Immersion Techniques (AIT) experiments, could only cause 10% mortality in LPTs [45]. Resende in [50] also shows that methanol, acetone, ethanol, and xylol cause no larval mortality against* Amblyomma cajennense* Fabricius, 1787 ticks. The difference in susceptibility of the different age groups of ticks to the solvents is attributed to the fact that adult ticks have a protective cuticle layer that is supposed to protect the tick from the effects of dehydration and other physical and chemical effects. This cuticle is also important in the reproduction processes of the tick because it is the site for pheromone production [51, 52]. The challenge is that, despite some organic solvents like acetone and chloroform being very good solvents to active components of plants, they can also dissolve the cuticle layer of ticks with fatal consequences of mortality and reduced reproductive capacity of the ticks [45]. The same can be said for other nonpolar solvents, for example, hexane and petroleum ether, but it must be noted that all solvents have differential effects on ticks depending on the capacity and concentrations of the solvents used [49, 53]. Tick larvae however do not have the cuticle layer and therefore do not suffer the same fate as adult ticks.

While organic solvents generally yield high active compounds, hexane in this study was ineffective. To confirm this assertion, there is need to compare the chemical yield extracted using the different organic solvents.

Organic solvents are generally superior for extraction of bioactive compounds [37, 54]. However, in the search for least cost animal health products for use by marginalised smallholder farmers, the use of organic solvents is limited by affordability and availability. There are also environmental and human health concerns worldwide over using organic solvents [55].

The absence of adverse clinical signs and behavioural change in the mice that received the different hot water leaf extract concentrations is a clear demonstration that use of this plant can be relatively safe. It does not, however, rule out health problems after repeated exposure to higher concentrations or misuse. Findings by Gakuya et al. in [33] showed five mortalities out of eight sampled mice in toxicity experiments that investigated the effect of aqueous* M. edulis* root-tuber extracts on nematodes in livestock using a 20 g/kg body mass concentration. Differences in the current experiment and the one conducted by Gakuya et al. may be attributed to the different plant parts used in the experiments. Bioactive compounds are more likely to be more concentrated in the roots than in the leaves.

## 5. Conclusion

*Maerua edulis* possesses acaricidal properties that potentially match those of amitraz-based Tickbuster when extracted with the suitable solvents and when applied at the correct concentrations. Preparing the plant material by soaking in hot water and adding 1% v/v surfactant improves effectiveness and is an easier way of optimizing activity of this plant for controlling ticks. The method is suitable for use by resource-poor farmers in developing countries because it promises to be both effective and much more economical than using organic solvent extraction. Further work is required to determine the efficacy of the same treatments against adult ticks.

## Figures and Tables

**Table 1:** A summary of experimental treatments used in the study.

Type of solvent used	Description of treatments
Cold water only	5, 10, 20, 25% v/v serial dilutions of cold water extracted *M. edulis*
Cold water plus surfactant	5, 10, 20, 25% v/v serial dilutions of cold water extracted *M. edulis* and 1% v/v surfactant
Hot water plus surfactant	5, 10, 20, 25 v/v serial dilutions of hot water (70°C) extracted *M. edulis* and 1% v/v surfactant
Hexane	5, 10, 20, 25 v/v serial dilutions of hexane extracted *M. edulis*
Methanol	5, 10, 20, 25% v/v serial dilutions of methanol extracted *M. edulis*

Positive control: Tickbuster prepared at 0.2% v/v with water.

Negative controls: Either distilled water, distilled water plus 1% surfactant, methanol, or hexane.

**Table 2:** Comparison of least square mean (LSM) mortalities of tick larvae from cold water plus surfactant and hot water plus surfactant extracted *M. edulis* treatments.

Treatment	Concentration (%)	Mortality ranks	
		24 h	48 h
Cold water + surfactant	5	54.1^a^	73.1^a^
	10	62.0^a^	46.7^a^
	20	73.9^a^	47.0^a^
	25	63.9^a^	72.1^a^

Hot water + surfactant	5	128.9^b^	107.4^b^
	10	132.7^b^	120.8^b^
	20	132.0^b^	113.5^b^
	25	138.9^b^	113.0^b^

Note: within a column, means with different superscripts differ significantly (*P* < 0.05).
